# Spatiotemporal Dynamics of Ecological Vulnerability to Climate Change in Northwestern Sichuan’s Terrestrial Ecosystems of China: Conservation Implications

**DOI:** 10.3390/biology14111625

**Published:** 2025-11-19

**Authors:** Cuicui Jiao, Xiaobo Yi, Ji Luo, Ying Wang, Yuanjie Deng, Jiangtao Gou, Danting Luo

**Affiliations:** School of Economics, Sichuan University of Science & Engineering, Yibin 644000, China; abobobi@163.com (X.Y.); steelseek@suse.edu.cn (J.L.); hywangying@suse.edu.cn (Y.W.); ecodyj@suse.edu.cn (Y.D.); 18227618298@163.com (J.G.); 13683404454@163.com (D.L.)

**Keywords:** exposure–sensitivity–resilience framework, ecological protection, ecological vulnerability, dynamic assessment, Northwestern Sichuan, terrestrial ecosystems

## Abstract

Climate change intensifies ecosystem vulnerability in mountainous regions, particularly in Northwestern Sichuan’s Terrestrial Ecosystems (TENS), where complex terrain amplifies impacts on biodiversity and carbon dynamics. This study assesses spatiotemporal ecological vulnerability using the IPCC exposure-sensitivity-resilience framework. We applied autoregressive modeling and a 5-year moving window to monthly NDVI, temperature, and precipitation data from 1983 to 2022. Results show vulnerability index (VI) increases latitudinally from south to north, driven by inverse temperature correlations. Longitudinally, VI forms a V-shaped pattern due to topographic and monsoon influences. Wetlands are most vulnerable (VI ≈ 0.48) from precipitation sensitivity, while forests show lowest vulnerability (VI ≈ 0.43) due to high resilience. Temporally, VI fluctuates nonlinearly with decline (1985–1994) under cool-humid conditions, increase (1994–2008) amid warmer-drier El Niño effects, and sharp decline (2008–2011) from La Niña and sand control initiatives. Spatially, 34.6% of areas exhibit decline-increase-decline-increase trends. Centroids of decreasing VI shift southwest-to-north, indicating recovery diffusion. Increasing VI centroids move northwest-central-north. These findings underscore ecosystem-specific adaptive management and conservation policies, especially in northern TENS, to mitigate accelerating climate pressures.

## Simple Summary

Climate change is exerting mounting pressure on terrestrial ecosystems in Northwestern Sichuan (TENS) of China, where rugged topography and variable climate render them particularly vulnerable. Previous studies have subsumed TENS within the broader analyses of the Tibetan Plateau, thereby overlooking its dynamics and heterogeneities. Consequently, the spatiotemporal variations in TENS’ vulnerability remain poorly understood. This study aims to demonstrate how vulnerability varies across spaces and over time, identify ecosystem-specific vulnerabilities, phased interannual dynamics, trend conversions, and migration paths of vulnerability changing trends. Using data on vegetation growth, temperature, and precipitation, we found that vulnerability increases from south to north and forms a V-shape from west to east. Wetlands are the most vulnerable, while forests are more resilient. Over time, vulnerability dipped in cool, wet periods, rose during warmer, drier spells linked to weather events, and fell sharply with wetter conditions and anti-desert efforts. About a third of the area showed back-and-forth trends, with recovering spots spreading southwest to north and worsening ones moving northwest to the center, then north. These insights call for focused protection and tailored approaches for each ecosystem type, helping society build adaptive plans to safeguard biodiversity and support sustainable land use in similar vulnerable regions worldwide.

## 1. Introduction

Climate change has emerged as one of the most pressing environmental challenges of the 21st century. It profoundly impacts terrestrial ecosystems through alterations in temperature, precipitation patterns, and extreme weather events [1,2]. These changes have exacerbated ecological stressors, leading to shifts in biodiversity, ecosystem structures, functions, and services [3]. Such impacts are particularly evident in high-altitude and mountainous regions [4]. Within this broader context, the assessment of ecological vulnerability to climate change has emerged as a critical subfield in environmental science [5,6]. Ecological vulnerability is defined as the susceptibility of ecosystems to harm from climate-induced disturbances [6]. In previous studies, it has been quantified or operationalized through frameworks that integrate multiple indicators, such as exposure, sensitivity, and adaptive capacity [2]. The terrestrial ecosystems of Northwestern Sichuan (TENS) are a mountainous transitional zone between the Tibetan Plateau and Sichuan Basin. It features complex topography and diverse ecosystems. At such, TENS represents a critical hotspot facing heightened risk from climate variability [7]. Understanding the spatiotemporal dynamics of the ecological vulnerability is essential. This knowledge informs targeted conservation strategies, particularly amid accelerating climate impacts in fragile environments [5].

The existing literature on ecological vulnerability assessment has evolved into diverse frameworks and approaches. For instance, risk–based frameworks integrate hazard probability and impact magnitude, making them suitable for short-term disaster forecasting (e.g., in flood–prone areas). However, these frameworks often overlook long-term adaptive processes [8]. Indicator–based approaches aggregate multidimensional metrics such as biodiversity and soil degradation, excelling in broad-scale evaluations. Nevertheless, they might suffer from subjectivity in indicator selection and weighting [9]. Dynamic simulation models, including agent–based or process–based simulations, are ideal for scenario projections. Yet, they require extensive computational resources and validation data, limiting their use to some degree [10].

The IPCC framework conceptualizes ecological vulnerability as a function of exposure, sensitivity and resilience. Exposure pertains to the degree of external climatic disturbances that an ecosystem encounters. Sensitivity refers to the inherent susceptibility of the system to these disturbances. Resilience indicates the capacity to recover and maintain function. This framework offers a comprehensive and interdisciplinary approach. It emphasizes extrinsic stressors (exposure), intrinsic ecosystem responses (sensitivity), and recovery potential (resilience). As a result, it is particularly applicable to climate-driven vulnerability assessments in diverse landscapes [2]. The IPCC framework has significantly advanced vulnerability science by integrating climatic stressors with ecosystem responses. Building on these global insights, some studies at broad scales have progressed from identifying exposure as the dominant global driver, with sensitivity and resilience modulating regional variations. For example, Li et al. found that global vulnerability patterns are largely determined by exposure, with 61.31% of terrestrial vegetated areas capable of mitigating climate impacts, concentrated in polar regions, boreal forests, tropical rainforests, and intact forests, and a highly significant negative correlation between exposure and sensitivity [11]. In contrast, other research efforts have adopted innovative climate variables, such as niche novelty encompassing temporal, spatial, and ecological mismatches. Kling et al. elucidated patterns of vegetation vulnerability over extended periods, revealing that novel climates could lead to complex biogeographic responses and non-analog communities [12].

Transitioning to regional levels, advancements include the use of vegetation indices and dynamic models for spatiotemporal assessments. For example, Sang and Hamann mapped aridity vulnerability in North America, identifying climatic limiting factors through remote-sensing-based analysis [13]. Similarly, Xu et al. projected expansions of high-vulnerability areas in Southwest China under future scenarios, based on climate exposure, vegetation stability, and productivity [14]. Cai et al. revealed slight declining vulnerability trends in arid Northwest China, with vulnerability exhibiting a significant “stepped” differentiation where eastern and western regions were markedly lower than the central region, and the gravity center shifting in a “Z” direction [15]. These studies all highlight the influence of environmental drivers on vulnerability trends.

Furthermore, applications of geodetector analyses on the Tibetan Plateau have underscored environmental drivers over socioeconomic factors. Li and Song found that natural factors, with q-statistical values ranging from 0.036 to 0.918 (average 0.449), dominated spatiotemporal variations more than socioeconomic factors (average 0.051) [16]. This is comparable to Northwestern Sichuan’s plateau environment, emphasizing the primacy of natural influences in high-elevation ecosystems. Drought assessments in Mongolian grasslands have revealed increasing vulnerability correlated with heightened drought frequency. Nandintsetseg et al. identified higher risks in northcentral and northeast Mongolia [17]. In Northwestern Sichuan, investigations focused on geological factors have identified spatial differences influenced primarily by variations in the frequency of geological disasters. Xiao et al. showed vulnerability increasing from 2010 to 2020 but improving slightly in the latter five years, with nonlinear but significant geological influences [18]. These studies suggest the need for strategic management for adaptation and ecosystem conservation.

Despite advancements in scale, methodology, and integration of drivers, several key limitations remain. These include: (1) The research has placed limited focus on transitional zones with complex terrain, which might experience amplified climate change impacts. That may potentially result in overlooked nonlinear vulnerability patterns. (2) The research has provided inadequate exploration of long-term dynamics, especially phase-specific trend reversals. (3) The research has overlooked the migratory patterns in vulnerability trends, which are crucial for comprehending ecosystem responses across time and space. This further impedes the formulation of adaptive management strategies.

The TENS is located on the eastern margin of the Tibetan Plateau. That exemplifies these gaps and underscores the urgent need for refined vulnerability assessments, given its dramatic elevational gradients (~750–6500 m), interactions between monsoonal and westerly climates, and intensive agro-pastoral activities [7]. As a vital ecological barrier for the headwaters of the Yangtze and Yellow Rivers, TENS is an important component of China’s “Two Screens and Three Belts” national ecological security strategy—an integrated conservation plan designed to preserve biodiversity and mitigate environmental risks in ecologically fragile zones. Accordingly, the TENS has received substantial government emphasis and support through initiatives like ecological redlines and restoration programs aimed at combating climate-induced degradation [19,20]. Despite its ecological significance, prior studies have often subsumed TENS within broader Tibetan Plateau analyses, neglecting its unique dynamics and disparities [16]. This oversight may result in incomplete vulnerability profiles, potentially leading to underestimating risks in high-vulnerability zones like northern wetlands and mid-elevation grasslands, while also overlooking opportunities for resilience-building interventions.

To address these gaps, this study employs a comprehensive vulnerability assessment model adapted from Li et al. [11]. The model incorporates exposure, sensitivity, and resilience indices. We apply an autoregressive model and a 5-year moving window approach to the datasets of Normalized Difference Vegetation Index (NDVI), temperature, and precipitation spanning from 1983 to 2022. The primary objectives are to (1) elucidate spatial heterogeneity and ecosystem-specific vulnerability in transitional zones with complex terrain, addressing the overlooked nonlinear patterns; (2) delineate phased interannual dynamics and trend reversals, filling the gap in long-term exploration; (3) reveal migration paths of vulnerability changing trends to derive conservation implications, enhancing understanding for adaptive management. Ultimately, these efforts provide a robust basis for adaptive strategies in TENS and similar regions.

## 2. Materials and Methods

### 2.1. Study Area

The TENS region comprises the Ganzi Tibetan Autonomous Prefecture and the Aba Tibetan and Qiang Autonomous Prefecture in Sichuan Province. It is located in the Hengduan Mountains to the east of the Tibetan Plateau (31°52′–34°20′ N, 97°21′–104°27′ E). Based on the land-cover data of 2020 from Yand and Huang [21], excluding water bodies, snow and ice, bare land, and impervious surfaces, the total area of TENS is approximately 236,000 km^2^.

**Figure 1:**
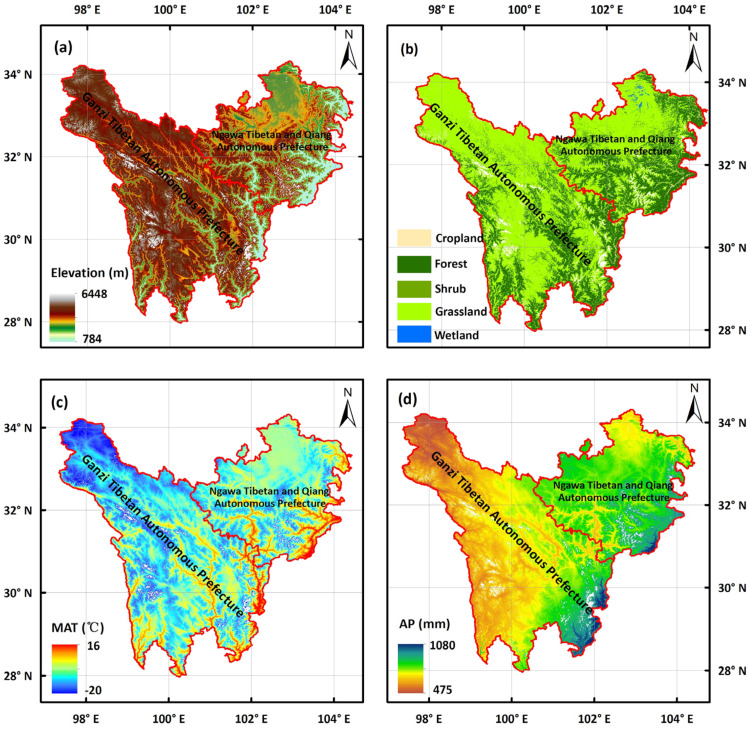
General geographic information of the TENS region, including (**a**) spatial distribution of the elevation; (**b**) Spatial distribution of land-cover types; (**c**) Spatial distribution of mean annual temperature (MAT); and (**d**) Spatial distribution of annual precipitation (AP). The white areas in this figure represent the Nodata regions, which refer to non-vegetation types including water bodies, snow and ice, bare land, and impervious surfaces.

The terrain slopes from northwest to southeast over a complex geomorphic landscape and lies at an elevation between 3500 and 4500 m (Figure 1a). The region contains five main ecosystem types: croplands, forests, grasslands, shrublands, and wetlands [21]. The grassland ecosystem covers the largest area, accounting for 63.82% of TENS, followed by the forest ecosystem, which occupies 31.76% of the region [21] (Figure 1b). The TENS regional climate is characterized by long, cold winters with no distinct summer, but relatively temperate and cool conditions. The annual average temperature ranges from −22 °C to 16 °C (Figure 1c), and the annual precipitation ranges from 475 mm to 1080 mm [22] (Figure 1d).

### 2.2. Methods

#### 2.2.1. Data Sources and Preprocessing

- Data Sources

The data used in this study included the Normalized Difference Vegetation Index (NDVI), temperature, precipitation, and land-cover data. The NDVI data from 1983 to 2022 were sourced from the PKU GIMMS NDVI dataset (version 1.2) provided by Peking University (https://zenodo.org/records/8253971, accessed on 15 January 2025) [23]. The PKU GIMMS NDVI (version 1.2) was developed to improve the temporal consistency of long-term vegetation indices by addressing artifacts from NOAA satellite orbital drift and AVHRR sensor degradation. The original data have a temporal resolution of 15 days and a spatial resolution of 1/12° (~8 km), which were generated using biome-specific backpropagation neural network (BPNN) models trained on the GIMMS NDVI3g product and 3.6 million high-quality Landsat NDVI samples. To extend coverage to 2022, MODIS NDVI (MOD 13C1) data were integrated using a pixel-wise Random Forest fusion approach. The final product exhibits improved temporal consistency and accuracy, validated against Landsat NDVI [23].

The monthly precipitation and mean temperature data from 1983 to 2022 were obtained from the National Earth System Science Data Center (https://www.geodata.cn/main/face_science_detail?guid=192891852410344&typeName=face_science, https://www.geodata.cn/main/face_science_detail?guid=164304785536614&typeName=face_science, accessed on 15 January 2025) with a spatial resolution of 1 km [22]. This dataset was generated through spatial downscaling over China using the Delta method, based on the global 0.5° climate dataset released by the Climatic Research Unit (CRU) and the high-resolution global climate dataset provided by WorldClim. The reliability of the downscaled dataset was validated using observations from 496 independent meteorological stations, demonstrating credible results [22].

The land-cover data were from the 30 m annual China Land Cover Dataset (CLCD) developed by Yang and Huang [21]. This dataset includes 1985 and annual data from 1991 to 2022 (https://zenodo.org/records/12779975, accessed on 15 January 2025). This dataset was generated on the Google Earth Engine platform utilizing 335,709 Landsat images. Training samples were derived from stable samples within China’s existing Land-Use/Cover Datasets (CLUD), combined with visually interpreted samples from satellite time-series, Google Earth, and Google Maps. Multi-temporal metrics constructed from all available Landsat data were classified using a random forest algorithm. The resulting classifications were enhanced for spatial-temporal consistency through a dedicated post-processing step incorporating spatial-temporal filtering and logical reasoning [21].

- Data preprocessing

To minimize abnormal fluctuations caused by cloud cover or other weather-related factors, the maximum value composite (MVC) method was employed to aggregate biweekly NDVI data to a monthly scale. For pixels with no data, a 3 × 3 neighborhood averaging approach was employed for gap filling. To match the spatial scale required for detailed vulnerability assessments and conservation planning, we resampled the ~8 km PKU GIMMS NDVI data to a 1 km resolution using the bilinear interpolation method. While resampling may introduce some uncertainty, it is a necessary step for integrating multi-source data and is widely applied [24]. This approach ensures spatial consistency across multiple datasets (NDVI, precipitation and temperature) and allows for a finer-grained analysis of ecological vulnerability. This is critical for identifying localized hotspots relevant to management interventions.

In addition, to facilitate the analysis of temporal trends in vulnerability and to minimize uncertainties in the assessment model caused by extreme climatic or other sudden events, the moving window approach was adopted. The principle of this method is to divide the entire dataset into relatively small, continuous time segments by setting a fixed unit interval (i.e., window) and moving it step by step across the series. This approach is commonly employed in long-term change research of vegetation growth [24,25,26]. In this study, monthly NDVI, temperature, and precipitation data from 1983 to 2022 were analyzed using a 5-year moving window length and 1-year step size, starting from 1983 and moving forward annually to end in 2022, to generate 36 overlapping periods of 1983–1987, 1984–1989, ……., 2018–2022, every period containing 60 months. The 5-year window was chosen as it is long enough to buffer the effects of short-term climate oscillations (e.g., El Niño-Southern Oscillation) and anomalous years, thus capturing more stable ecosystem responses to climate trends [24,26]. At the same time, it is short enough to detect decadal-scale shifts in vulnerability, providing a balance between signal stability and temporal resolution [24,26].

#### 2.2.2. Ecological Vulnerability Assessment Model

- Overview of the ecological vulnerability

In this study, the ecological vulnerability to climate change was assessed within a widely used framework of exposure, sensitivity, and resilience. Exposure refers to the extent of climate disturbance that terrestrial ecosystems may undergo [27]. Sensitivity refers to the extent to which an ecosystem may be affected under a certain disturbance [5,28]. Resilience refers to the capacity of an ecosystem to return to its original state after a disturbance [29]. The assessment model for ecological vulnerability was developed as follows (Equation (1)), by incorporating these three components into an overall vulnerability index, as proposed by Li et al. [11].

(1) $$VI=\sqrt{\frac{EI\times SI}{1+RI}}$$

Wherein, VI is the Ecological Vulnerability Index, EI is the Exposure Index, SI is the Sensitivity Index, and RI is the Resilience Index. This model integrates the three core components of vulnerability. EI and SI represent the potential impact, while RI acts as a mitigating factor. Vulnerability increases with greater exposure and sensitivity but decreases with higher resilience. This formulation ensures that resilience alone cannot eliminate vulnerability unless the initial impact (EI or SI) is zero. The final VI represents a synthesized measure of the ecosystem’s susceptibility to harm from climate change. It thus reaches its peak when resilience is absent, given the function of EI and SI [30]. Its value has been demonstrated to be effective in identifying vulnerable areas in previous studies [11,31,32].

- Resilience, Sensitivity and Exposure

Vegetation dynamics show long-term memory, influenced by current climate and past states, with ecosystems exhibiting persistence [33]. Accordingly, resilience can be quantified via autoregressive (AR) coefficients associated with vegetation restoration time and persistence [34], derived from prior fittings [35]. We applied an AR (1) multiple linear regression within each 5-year moving window (60 months) to characterize ecosystem responses to climate change at the grid scale (Equation (2)):

(2) $${NDVI}_{t}=\alpha \times {Temp}_{t}+\beta \times {Pre}_{t}+\gamma \times {NDVI}_{t-1}+\varepsilon_{t}$$

where ${NDVI}_{t}$ and ${NDVI}_{t-1}$ are the monthly standardized NDVI anomalies at time t and t−1. ${Temp}_{t}$ and ${Pre}_{t}$ are the monthly standardized temperature and precipitation anomalies at time t. α, β, and γ are the fitting coefficients. $\varepsilon_{t}$ is the residual term. To ensure comparability between model coefficients, monthly NDVI, temperature, and precipitation data within each 5-year moving window were standardized using the z-score method [28]. The coefficients α, β, and γ were normalized a 0–1 range using min-max scaling. That could ensure scale-invariant indices without bias from variable magnitudes.

The coefficients in the AR (1) model (Equation (2)) are intrinsically linked to the three dimensions of ecological vulnerability. The parameter γ captures the degree of dependence of current vegetation on its immediate past state, and thus reflects the memory effect of the ecosystem. Consequently, the Resilience Index (RI) can be defined as follows (Equation (3)) [33]:

(3) RI=1−γ

where RI represents the Resilience Index, and γ is the approximate value of ${NDVI}_{t-1}$ in Equation (2).

The parameters α and β quantify the magnitude of vegetation response to instantaneous temperature and precipitation changes. Higher absolute values indicate lower resistance to climate fluctuations. Thus, the Exposure Index (EI) can be defined as follows (Equation (4)) [31,32]:

(4) EI=α+β

where EI is the Exposure Index, α and β are the fitting coefficients of temperature and precipitation anomalies in Equation (2).

Sensitivity Index (SI) can be obtained by the weighted summation of the standardized meteorological anomalies and related fitting coefficients [28], as follows (Equation (5)):

(5) $$SI=\alpha \times T_{-norm}+\beta \times P_{-norm}$$

where SI is the Sensitivity Index, α and β are the fitting coefficients of temperature and precipitation anomalies in Equation (2). $T_{-norm}$ and $P_{-norm}$ represent the mean normalized temperature and precipitation within each 5-year window, respectively.

Based on a 1 km × 1 km grid cell scale, the EI, SI, RI, and VI were calculated using Equations (1)–(5) for each sliding window (1983–1987, 1984–1988, …, 2018–2022). The midpoint year of each window (1985, 1986, …, 2020) was used as the temporal reference, resulting in continuous time series data for each index from 1985 to 2020. These calculations were performed using R version 4.4.1 [36].

It is important to note the assumptions of the AR (1) model. This model assumes a linear relationship between vegetation anomalies and climate factors. It also assumes the time series within each 5-year window are stationary. While these assumptions simplify a complex reality, this approach is widely validated for capturing first-order ecosystem responses to climate variability over large scales [28,31,33]. The use of standardized anomalies helps to meet the stationarity assumption. The residual term $\varepsilon_{t}$ accounts for unexplained variance. Potential limitations include nonlinear responses and the influence of un-modeled drivers (e.g., CO_2_ fertilization, human activities). Those are acknowledged as sources of uncertainty in the interpretation of our results.

#### 2.2.3. Statistical Analysis Methods

To explore the zonal patterns of VI, we uniformly sampled 2088 points across the TENS. Sample points were selected using a systematic grid-based sampling scheme to ensure an even spatial distribution across the main ecosystem types. This sample size provided sufficient replication within each ecosystem for robust statistical comparisons, while remaining computationally efficient. The multi-year average values of 1985–2020 for VI, EI, SI, and RI at each sample point were extracted. Based on these data, one-way analysis of variance (ANOVA) with Tukey–Kramer test was employed to examine differences in VI and its components among five major ecosystems using IBM SPSS Statistics version 27.0 [37]. To better analyze the zonal regularity of VI, we aggregated the extracted values into spatial bins: 1° intervals for both latitude and longitude, and 100 m intervals for elevation. Within each bin, we calculated the mean values of VI, EI, SI, and RI. Subsequently, regression analyses (linear or piecewise linear regressions) were conducted on these binned means to quantify the relationships between each index and the geographic variables—latitude, longitude, and elevation. These analyses were conducted in R version 4.4.1 [36].

To uncover the temporal dynamics of VI, we calculated the yearly zonal mean values of VI, EI, SI, and RI for TENS from 1985 to 2020. Based on the yearly zonal mean values of VI, interannual variations were analyzed by using the piecewise linear regressions. These analyses identified the inflection points, where a significant change in the VI trend occurs, often leading to a new direction in the trend. According to the inflection points, the change trends of VI were classified into different stages. We further applied the centroid shift model to examine the trajectory of VI trend changes over the various stages. The centroid shift model calculates the geometric center (centroid) of a spatial distribution using weighted average coordinates (e.g., latitude and longitude) for each phase. By tracking the migration of these centroids over time, the model could characterize the temporal evolution of vulnerability changing trend, revealing directional shifts. This approach has been widely applied in ecology and geography to analyze temporal changes in spatial patterns [38]. The linear and piecewise linear regressions, as well as the centroid shift model, were implemented in R version 4.4.1 [36]. The spatial pattern maps were plotted using ArcGIS Desktop version 10.8 [39].

These spatial and temporal analyses are directly linked to conservation implications. By identifying the geographic hotspots of high vulnerability and understanding the temporal trends, our results can directly inform adaptive management strategies like corridor establishment or restoration priorities.

Notably, the distribution of ecosystems changed between 1985 and 2020. To avoid confounding the effects of climate change with those of land-use change, our primary analysis focused on areas where the five major ecosystem types remained stable throughout the 36-year period. This approach allows us to isolate the climate-driven vulnerability of established ecosystems. While this excludes areas of land-use transition, which are themselves important, it ensures that our findings on the vulnerability are not biased by the signals of conversion.

## 3. Results

### 3.1. Spatial Distribution Characteristics of TENS Vulnerability

#### 3.1.1. Spatial Patterns of VI

**Figure 2:**
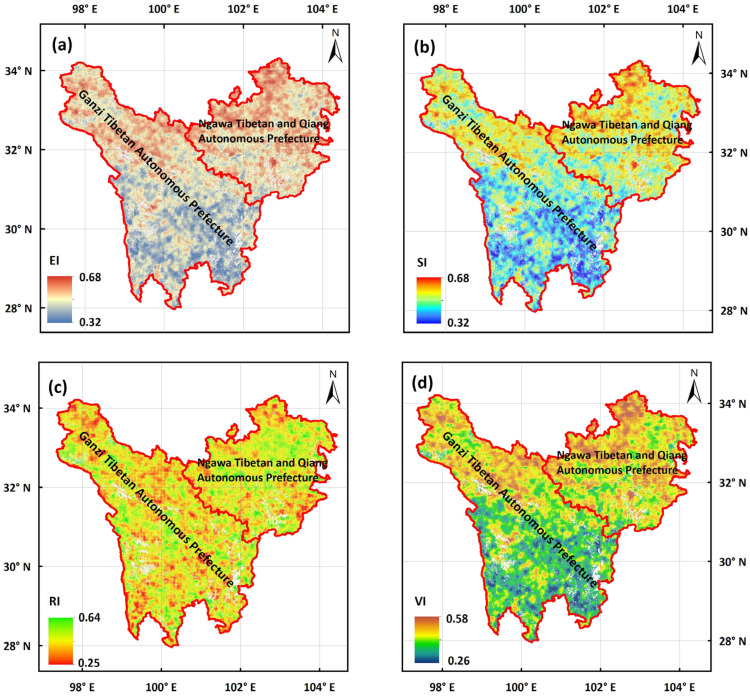
Spatial distributions of mean ecological vulnerability index (VI) and its three components (**a**) Exposure Index (EI), (**b**) Sensitivity Index (SI), and (**c**) Resilience Index (RI)—along with (**d**) overall VI, from 1985 to 2020. White areas denote no-data regions, which refer to non-vegetation types including water bodies, snow and ice, bare land, and impervious surfaces.

Based on the annual assessment results, we derived the spatial patterns of the multi-year averages of VI and its three components from 1985 to 2020 (Figure 2a–d). These indices exhibited significant spatial heterogeneity across the TENS. EI values increased from 0.32 in the south to 0.68 in the north (Figure 2a). SI showed a spatial pattern similar to EI (Figure 2b). In contrast, the RI values varied from 0.25 to 0.64 across the entire region (Figure 2c). The final spatial pattern of the VI was determined by the interplay of these three components. Notably, the RI partially counteracted the high exposure and sensitivity in certain areas, thereby mitigating overall vulnerability. Consequently, the comprehensive VI values ranged from 0.26 in the south to 0.58 in the north, indicating a clear north–south vulnerability gradient (Figure 2d).

#### 3.1.2. Zonal Characteristics of VI

**Figure 3:**
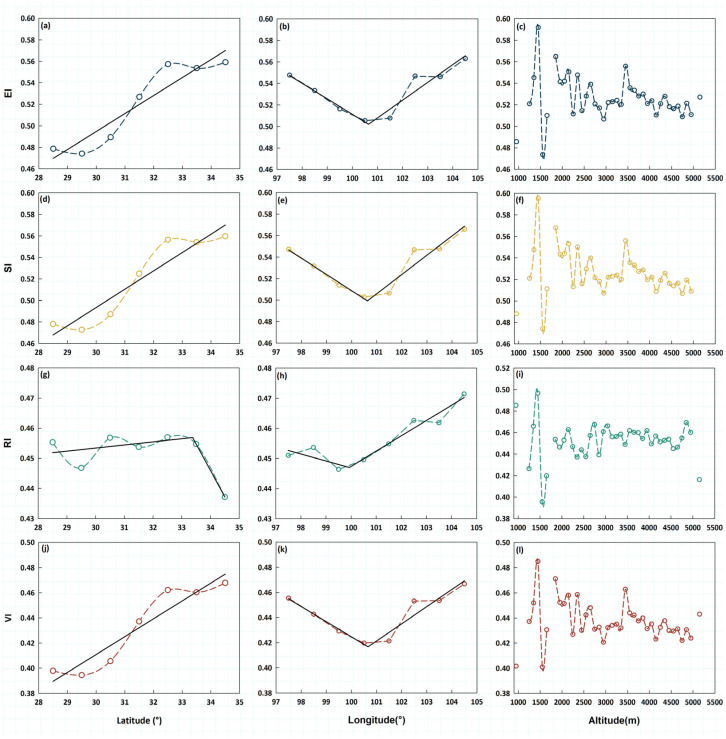
Variations in VI and its components EI, SI, and RI with latitude, longitude, and altitude. (**a**–**c**) EI; (**d**–**f**) SI; (**g**–**i**) RI; (**j**–**l**) VI. Left column: versus latitude; middle column: versus longitude; right column: versus altitude.

VI and its three components—EI, SI, and RI—exhibited distinct spatial gradients across latitudes, longitudes, and altitudes within the TENS. EI increased linearly with latitude (Figure 3a). Longitudinally, EI declined to a minimum near 100–101° E before a slight rise (Figure 3b). Altitudinally, EI generally decreased, with notable fluctuations (Figure 3c). SI showed broadly similar patterns with EI, increasing steadily with latitude (Figure 3d). Longitudinally, SI displayed a V-shaped pattern, declining to a minimum near 100–101° E followed by a modest increase (Figure 3e). Altitudinally, SI declined, with intermediate variability (Figure 3f). RI increased with latitude to a peak near 33–34° N before declining (Figure 3g). Longitudinally, RI decreased to a minimum around 99–100° E, followed by stabilization or a slight increase (Figure 3h). Altitudinally, RI declined, accompanied by irregular fluctuations (Figure 3i). The composite VI increased linearly with latitude (Figure 3j). Longitudinally, VI followed a V-shaped trajectory, declining to a minimum around 100–101° E before a slight increase (Figure 3k). Altitudinally, VI displayed substantial fluctuations, exhibiting no discernible regularity (Figure 3l). The analysis based on 2088 sampling points also generated similar results (Figure S1).

#### 3.1.3. Comparative Analysis of VI Across Ecosystem Types

**Figure 4:**
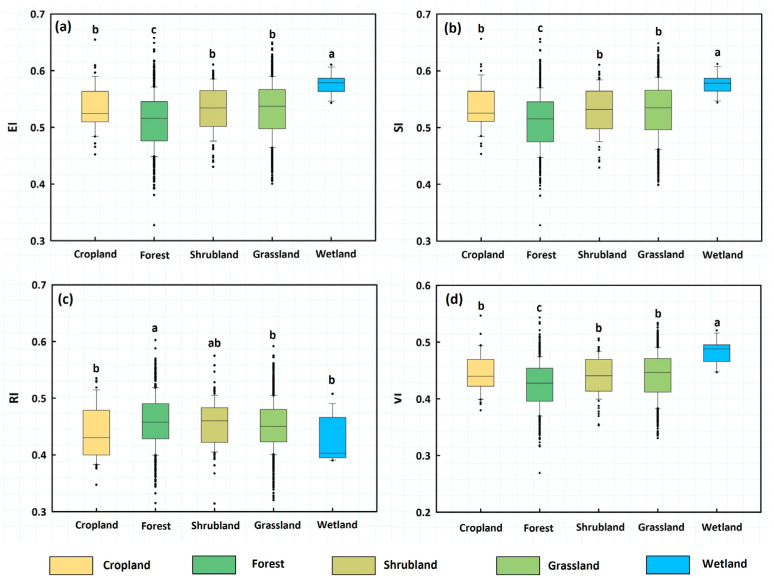
Differences in VI and its three components across different ecosystem types. (**a**) EI; (**b**) SI; (**c**) RI; and (**d**) VI. Different letters (a, b, c, etc.) denote significant differences in VI and its three components at *p* < 0.05 (Tukey–Kramer test).

Significant differences in VI were observed across the five main ecosystem types: forest, shrubland, grassland, cropland, and wetland (Figure 4). The differences in EI and SI among these ecosystems were consistent with those for VI (Figure 4a,b,d). For RI (Figure 4c), forest ecosystems exhibited the strongest resilience (RI = 0.458), slightly higher than those of shrubland ecosystems (RI = 0.456). Wetland ecosystems showed the lowest resilience (RI = 0.43), and grassland ecosystems showed an RI of 0.45. These values were significantly lower than those of forests but not significantly different from those of shrublands, croplands, and wetlands. Cropland resilience was significantly lower than that of forests and shrublands but not significantly different from that of grasslands and wetlands.

Wetlands were the most vulnerable ecosystem (VI = 0.48), considerably more fragile than the other ecosystems, according to VI (Figure 4d). Forests, on the other hand, were considerably less vulnerable than other ecosystem categories and the least vulnerable (VI = 0.43). There were no significant differences between shrublands and grasslands, and cropland vulnerability (VI = 0.44) was significantly higher than that of forests but much lower than that of wetlands. Although the difference was negligible, grasslands showed somewhat greater vulnerability than shrublands.

### 3.2. Temporal Dynamics of TENS Vulnerability

#### 3.2.1. Interannual Variations in Vulnerability from 1985 to 2020

**Figure 5:**
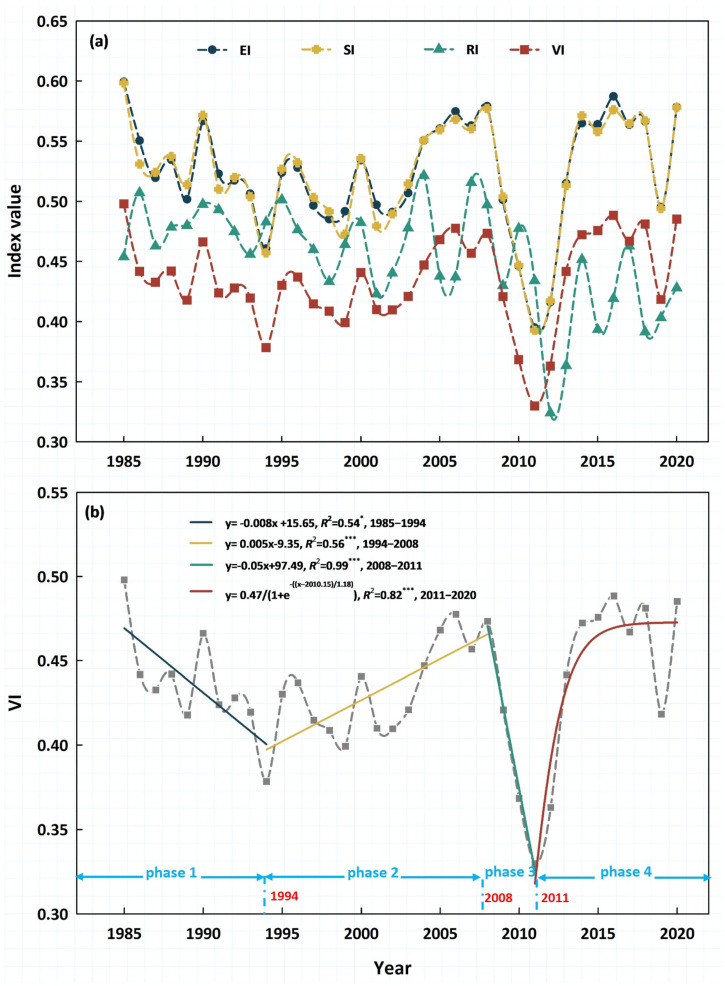
Dynamics of the regional average EI, SI, RI, and VI values from 1985 to 2020. (**a**) The annual values of EI, SI, RI, and VI. (**b**) Phase−specific dynamics in VI. * and *** indicate that the regression relationship was significant at the 0.05 and 0.001 level, respectively.

On average, the EI ranged from 0.39 (in 2011) to 0.60 (in 1985) in the TENS (Figure 5a), with a coefficient of variation (CV) of 8.97%. SI exhibited a similar temporal trend to EI, ranging from 0.39 to 0.60 (Figure 5a), with a CV of 9.00%. RI ranged from 0.32 (in 2012) to 0.52 (in 2004) (Figure 5a), with a CV of 9.44%. Ecosystem resilience (RI) partially buffered the combined effects of climate exposure and ecosystem sensitivity (SI), resulting in lower overall vulnerability, ranging from 0.33 (in 2011) to 0.50 (in 1985) (Figure 5a), with a CV of 8.75% (Figure 5a).

During the crucial transitions from 1985 to 2020, the regional average VI in TENS showed clear phase-specific dynamics, with inflection points in 1994, 2008, and 2011. The four phases of these dynamics were marked by a series of “significant decline–significant increase–sharp decline–stabilization” (Figure 5b). In particular, VI decreased at a pace of 0.008 annually between 1985 and 1994, increased at a rate of 0.005 annually between 1994 and 2008, and then dramatically decreased at a rate of 0.050 annually between 2008 and 2011. The VI showed an S-shaped trajectory from 2011 to 2020, with an initial increase and stabilization after 2014 (Figure 5b).

#### 3.2.2. Spatial Heterogeneities of Phase-Specific Interannual Variations in Vulnerability

**Figure 6:**
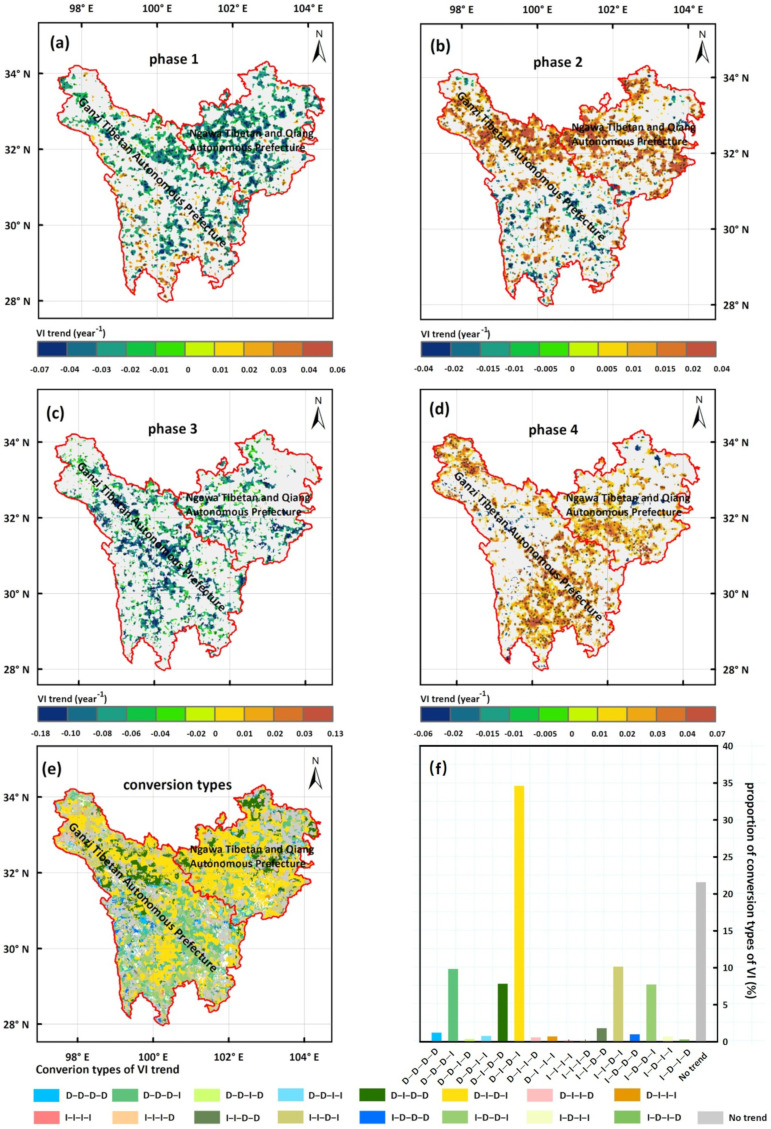
Spatial distribution of the phase-specific interannual trends of VI (**a**) during Phase 1 (1985–1994), (**b**) during Phase 2 (1994–2008), (**c**) during Phase 3 (2008–2011), and (**d**) during Phase 4 (2011–2020). Only areas with statistically significant trends (*p* < 0.05) were displayed in (**a**–**d**). Conversion types of VI trends during the period 1985–2020 (**e**). Area proportions of various VI trend conversion types (**f**).

The analyses mentioned above, based on regional averages, reflected overarching temporal shifts in VI while neglecting grid-level variability. However, individual grid cells may exhibit variable trends due to local heterogeneity. To better understand the spatiotemporal heterogeneity of VI trends and inform targeted conservation strategies (e.g., identifying regions of persistent vulnerability vs. recovery), we analyzed phase-specific interannual variations in VI by using linear regression at the grid-level during each phase identified from regional averages (1985–1994, 1994–2008, 2008–2011, and 2011–2020). To avoid overinterpreting weak trends or insignificant fluctuations, we only focused on statistically significant trends (*p* < 0.05, *t*-test) for increasing or decreasing VI. The spatial patterns of these significant interannual VI dynamics during the four phases are shown in Figure 6a–d.

- Phase 1 (1985–1994): Significant VI decreases (indicating reduced vulnerability and improved ecosystem quality) occurred in 27.76% of the TENS, particularly in western Aba Tibetan and Qiang Autonomous Prefecture. Significant increases (suggesting heightened vulnerability) were limited to 4.76% of the region. The majority (67.48%) showed no significant trend (Figure 6a, Table S1).
- Phase 2 (1994–2008): Significant VI increases (indicating increased vulnerability and ecosystem degradation) covered 31.43% of the region, mainly in northern Ganzi Tibetan Autonomous and Aba Tibetan and Qiang Autonomous Prefectures. Significant decreases were observed in 7.23% of the region. No significant trend dominated in 61.34% of the area (Figure 6b, Table S1).
- Phase 3 (2008–2011): Significant VI decreases (reflecting improved ecosystem quality) were widespread in 24.89% of the region. Significant increases were minimal, with small areas scattered throughout the northern Ganzi Tibetan Autonomous Prefecture and the Ganzi–Aba border. No significant trend was evident in 75.11% of the region (Figure 6c, Table S1).
- Phase 4 (2011–2020): Significant VI increases (indicating ecosystem degradation) occurred in 33.31% of the region, concentrated in central and southeastern TENS. Significant decreases (suggesting ecosystem improvement) were limited to 1.38% of the region, mainly in northern and western TENS. No significant trend prevailed in 65.31% of the area (Figure 6d, Table S1).

The increasing (I) and decreasing (D) trends of VI at each grid cell within the TENS were analyzed across the four distinct phases. These trends were then synthesized to identify complex conversion patterns from 1985 to 2020 (Figure 6e). Grid cells showing no significant changes in any of the four phases were classified as the “No trend” type. This category constituted 21.59% of the total TENS area (Table S2). The analysis revealed significant spatial heterogeneity in the conversion types. Several dominant patterns emerged, including D-I-D-I, I-I-D-I, D-D-D-I, I-D-D-I, and D-I-D-D, which collectively covered a substantial portion of the region. Among these, the D-I-D-I type was the most prevalent. It accounted for 34.62% of the TENS area, indicating a highly dynamic and fluctuating vulnerability landscape (Figure 6f, Table S2).

#### 3.2.3. Spatial Migration Dynamics of Vulnerability Changing Trends

**Figure 7:**
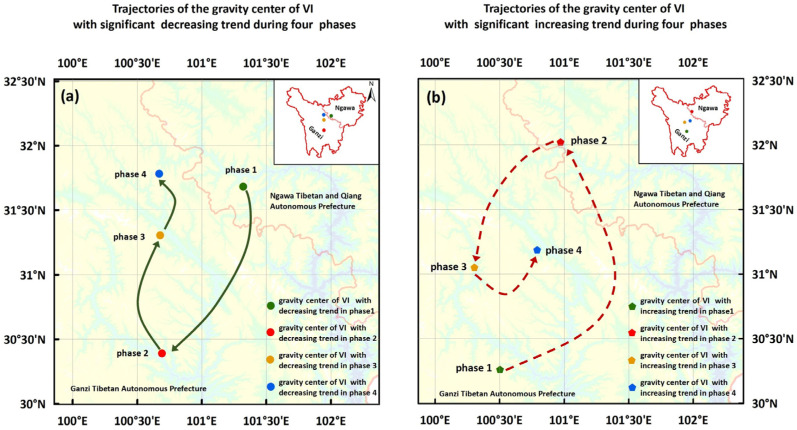
Centroid movement trajectories of VI with a significant decreasing trend (**a**) and with a significant increasing trend (**b**) during four different phases.

To further reveal the migration dynamics of vulnerability changing trend, which are crucial for understanding ecosystem responses to climate change and informing adaptive management, we applied a centroid shift model to areas with significant VI change trends (*p* < 0.05) across the four phases (Figure 7, Figure S2). Figure S2a–d illustrate the spatial distribution of areas exhibiting significant decreasing VI trends during these phases, indicating regions of continuous ecosystem recovery and enhanced stability. The centroid of these declining areas initially migrated southwestward from the central zone before shifting northward, reflecting a stability enhancement pattern characterized by ecosystem diffusion from central to peripheral regions (Figure 7a).

Conversely, Figure S2e–h present the spatial configuration of areas with significantly increasing VI trends across the four phases, suggesting heightened ecosystem vulnerability and substantial environmental stress in these locations. The centroid of these intensifying areas exhibited a three-stage movement: initial northwestward displacement, subsequent return to near-central positions, and final northward migration (Figure 7b). These shifts underscore the need for phase-specific interventions, such as in northern TENS, where increasing vulnerability has persistently migrated.

## 4. Discussion

Climate change poses profound threats to the Terrestrial Ecosystems of Northwestern Sichuan (TENS), a key subregion of the eastern Tibetan Plateau and a critical ecological barrier in western China [19,20]. This study addressed key gaps in understanding the ecological vulnerability of the TENS by employing the IPCC framework of exposure, sensitivity, and resilience, utilizing autoregressive modeling and a 5-year moving window approach on NDVI, temperature, and precipitation data from 1983 to 2022. Aligning with our primary objectives, we elucidated spatial heterogeneity and ecosystem vulnerability patterns, delineated phased interannual dynamics and trend conversions, and tracked the migration paths of vulnerability changing trend to inform conservation implications.

### 4.1. Spatial Heterogeneities and Ecosystem-Specific Vulnerabilities

This study revealed that the ecological vulnerability in TENS exhibits a clear latitudinal zonality. The VI increases from south to north, a spatial pattern consistent with similar observations on the broader Tibetan Plateau [16]. This gradient is primarily driven by climatic variations. Specifically, temperature shows a pronounced decrease with increasing latitude, which is inversely correlated with the VI (Figure S3). The northern regions of the TENS are characterized by higher altitudes. These areas face amplified exposure to climatic extremes, such as prolonged frost periods and intensified freeze–thaw cycles [40], which exacerbate ecosystem stress. This altitudinal effect compounds the latitudinal temperature decline, leading to several consequences. It elevates the EI, moderately increases the SI while reducing the RI [11,41]. Collectively, these factors shorten the growing season, constrain vegetation recovery, and ultimately reduce primary productivity [2].

Longitudinally, our analysis revealed a distinct V-shaped pattern in VI. Values decreased from the western edge toward 100–101° E and then increased eastward. This finding refines a previously reported pattern [18]. That study showed a generalized east-high-west-low gradient. Our work provides a more nuanced depiction of this longitudinal variation. The pronounced longitudinal zonality in VI is attributed to a complex interplay of topographic modulation and climatic transitions. This pattern aligns with a key climatic boundary at 100–101° E. Western ecosystems are shaped by South Asian monsoons and the westerlies (Figure S4). These influences foster relatively stable hydroclimatic conditions, despite overall aridity. In contrast, East Asian monsoons govern the eastern zones (Figure S4), introducing heightened variability in precipitation and temperature.

Topographically, the western highlands block moisture-laden westerly winds. This process creates rain shadows that cultivate arid yet climatically consistent environments [42]. This consistency reduces vulnerability. It diminishes both exposure to climate anomalies and ecosystem sensitivity. Conversely, eastern valleys and basins amplify monsoonal variability. This intensifies exposure to precipitation fluctuations and heightens ecosystem sensitivity. As a result, overall vulnerability is elevated. Central to this dynamic is a nonlinear vulnerability response to temperature. Temperature rises monotonically with longitude across the TENS (Figure S4). The VI, however, displays a V-shaped relationship with this temperature gradient. VI reaches its minimum at an intermediate thermal optimum (around 0–2 °C) near the 100–101° E meridian. It increases toward both the colder western and warmer eastern extremes (Figure S4). This suggests temperature extremes exacerbate vulnerability. Both cold and hot conditions create physiological stress for vegetation, such as cold limitation in the west and heat/drought stress in the east. Monsoon-driven climate anomalies further compound this stress.

Across ecosystem types, our findings show wetlands are significantly more vulnerable to climate change. In contrast, forests demonstrate comparatively lower vulnerability. This finding contrasts with prior research, such as global biomes-focused studies by Li et al. [11], county-scale analyses in arid areas of China by Cai et al. [15], and regional investigations in western Sichuan Mountain region by Xiao et al. [18]. Those studies often overlooked direct, comparative vulnerability assessments, particularly for wetlands. Wetlands’ heightened vulnerability stems from their intrinsic dependence on stable hydrological inputs. This reliance makes them highly susceptible to precipitation anomalies and altered water regimes [43]. In contrast, forests often exhibit greater resilience. They benefit from features like deeper root systems, greater structural diversity, and denser canopies. Higher biodiversity and stronger self-recovery capacities also help buffer climatic disturbances [44]. From a broader theoretical standpoint, this aligns with resilience theory [29]. This theory posits that a system’s intrinsic properties and memory modulate its response to extrinsic stressors [33]. Our model’s autoregressive coefficients partially capture this intrinsic memory. This perspective is also consistent with the IPCC’s framework [1], which emphasizes the critical role of adaptive capacity in determining overall vulnerability.

### 4.2. Interannual Dynamics of TENS Vulnerabilities

The interannual dynamics of the VI in the TENS exhibited a clear four-phase pattern between 1985 and 2020, reflecting nonlinear fluctuations driven by climatic variations, ecological processes, and policy interventions.

In the first phase (1985–1994), the VI showed a gradual decline, suggesting an improvement in ecosystem conditions. The decline in vulnerability was primarily attributed to favorable climatic conditions, notably from 1985 to 1991. During this period, the aridity index (AI) decreased, the humidity level rose, and temperatures cooled, leading to a reduction in drought stress (Figure S5), promoting ecosystem recovery. Even during 1991–1994, when AI slightly increased, temperatures continued to decrease (Figure S5), likely minimizing evapotranspiration and preserving soil moisture, which allowed ecosystems to further improve.

The second phase (1994–2008) marked a moderate increase in VI, signaling heightened vulnerability. This phase was characterized by a shift toward warmer and drier conditions (Figure S5), driven by a moderate El Niño event that occurred between September 1994 and March 1995 [45]. That intensified exposure to climate variability, exacerbating sensitivity, particularly in northern regions. This shift aligns with regional aridification trends, where prolonged warming has disrupted hydrological cycles, a phenomenon noted in other studies [46].

A sharp decline in VI occurred in the third phase (2008–2011), signaling significant improvements in ecosystem conditions. During this period, favorable climatic conditions were characterized by cooler temperatures and increased humidity (Figure S5). That resulted from a moderate La Niña event that occurred between August 2007 and May 2008 [45], mitigating the prior drought impacts and enhancing ecosystem productivity. Additionally, the initiation of sand control and desertification prevention projects of Northwest Sichuan in 2007 played a critical role [47]. These projects implemented measures such as grazing restrictions, afforestation, and soil stabilization in the northwestern regions, contributing to the substantial reduction in VI by restoring degraded land.

Post-2011, the VI exhibited S-shaped stabilization, with minor fluctuations. That suggests a balance between climatic pressures and protective interventions. Rising temperatures after 2011 (Figure S5) stemmed from a moderate El Niño event from June 2009 to April 2010 [45]. That initially contributed to a slight increase in vulnerability by enhancing EI and challenging ecosystem adaptability. However, the 2015 Ecological Civilization System Reform Plan introduced key institutional safeguards, including the establishment of ecological redlines and natural resource audits. Such steps curbed degradation and kept VI stable amid ongoing stressors.

This phased analysis advances prior research, by identifying key drivers and transitions that have often been overlooked in long-term or static assessments. For instance, Xu et al. emphasized climate exposure and vegetation productivity in Southwest China [14]. However, they overlooked historical phases and policy effects. Similarly, Pan et al. studied vulnerability in the Yangtze River Basin, focusing on habitat structure and function [48]. Yet, they missed key inflection points [48].

Spatial heterogeneity in vulnerability trends was evident at the grid level, with the D-I-D-I pattern being the most prevalent. This pattern highlights the reversible and oscillatory nature of ecological vulnerability, in line with resilience theory. This theory emphasizes that ecosystems can recover from periods of degradation if given sufficient time and favorable conditions [29]. Among the areas with the D-I-D-I pattern, approximately 85% were located in high-elevation zones characterized by medium to large mountain relief (Figure S6, Table S3). These complex terrains are particularly sensitive to climate fluctuations and human disturbance, leading to amplified responses in vulnerability dynamics. The pronounced topographic heterogeneity in these mountainous regions contributes to spatial differences in exposure, vegetation adaptability, and soil stability, which collectively shape the observed D-I-D-I patterns.

Vulnerability trend hotspots migrated northward, with centroids of decreasing VI moving southwest-to-north (indicating recovery diffusion), and increasing VI centroids shifting northwest-central-north (reflecting sustained stress). These movements are driven by climatic gradients, with northern areas experiencing elevated exposure to aridity (increasing EI). Additionally, human activities such as concentrated grazing in the north have contributed to these shifts. The observed recovery diffusion is likely a result of policy spillover, with forest restoration efforts in central areas extending ecological benefits to peripheral regions. Our phase-specific centroid tracking extends Zhang et al.’s work [31] in the Yellow River Basin, providing more detailed directional insights. However, it may not fully capture subtle shifts in areas without significant trends.

These spatiotemporal shifts further underscore the nonlinear responses of TENS vulnerability to management interventions and climatic extremes, building on the observed four-phase fluctuations and grid-level oscillatory patterns. For instance, management interventions such as grazing bans and ecological restoration projects (e.g., the 2007 sand control initiatives and 2015 Ecological Civilization reforms) elicited nonlinear improvements in VI, where initial gradual declines accelerated into sharp reductions (e.g., −0.050/yr in 2008–2011), reflecting ecosystem recovery once interventions surpassed critical disturbance levels, leading to enhanced lowered VI. Conversely, climatic events like extreme precipitation, embedded within the warmer-drier shifts (e.g., 1994–2008 phase with rising AI), triggered nonlinear spikes in VI, amplifying exposure and sensitivity in a V-shaped manner similar to the temperature response, where deviations from optimal hydroclimatic conditions caused abrupt increases in vulnerability (worsening ecosystem states) before stabilizing under subsequent favorable conditions or interventions. This nonlinearity highlights how small perturbations can lead to disproportionate VI changes, with recovery phases showing reversible declines (e.g., D-I-D-I patterns in high-elevation zones), offering new perspectives on vulnerability dynamics by revealing that targeted interventions can nonlinearly buffer against climatic extremes, promoting long-term stability despite oscillatory trends.

### 4.3. Policy and Management Recommendations

This study reveals pronounced spatial heterogeneity and temporal nonlinearity in the ecological vulnerability of TENS to climate change, driven by climatic gradients, topographic influences, and human activities. These findings underscore the need for adaptive, ecosystem-specific management strategies that integrate satellite-derived insights with on-ground interventions.

- Targeted conversation efforts in high-risk zones

Our study identifies the northern, high-altitude regions as having the highest ecological vulnerability due to prolonged frost and freeze–thaw cycles. Implement rotational grazing, seasonal bans, and grass seeding. Promote climate-smart agriculture to boost resilience and food security. Conservation resources should be prioritized for these areas. Specific measures should include promoting sustainable grassland management practices like rotational grazing, seasonal grazing bans, and artificial seeding.

- Adaptive management for ecosystem-specific vulnerabilities

Vulnerability varies significantly among ecosystem types. Therefore, management strategies must be tailored. For wetlands, priorities should include restoring natural hydrological rhythms and enhancing their buffering capacity against precipitation anomalies through engineering, such as water retention dams. For forests, their high resilience should be leveraged by establishing them as ecological barriers through targeted afforestation and biodiversity enhancement programs.

- Incorporating climatic variability into land use planning

The study reveals distinct longitudinal and latitudinal vulnerability gradients, strongly linked to climatic gradients, especially in transitional zones where monsoons converge. Consequently, land use planning must integrate climate change projections. Climate variability should be explicitly incorporated into land-use zoning regulations in these transitional zones, with restrictions on high-intensity agriculture. Urban and rural development policies should also promote low-carbon infrastructure and green spaces to mitigate further stress on vulnerable ecosystems.

- Enhancement of ecological monitoring and data collection

Ecological vulnerability in TENS is dynamic, with a shifting trend northward. To manage this effectively, a comprehensive monitoring network combining remote sensing and ground-based observations is essential for real-time tracking of ecosystem health. This system should facilitate data sharing and collaboration among managers, researchers, and local communities, ensuring that conservation actions can respond swiftly to spatiotemporal vulnerability dynamics.

- Policy integration and multi-stakeholder involvement

Our research confirms that policy interventions, such as the “Ecological Civilization System Reform”, have positively contributed to stabilizing regional ecological vulnerability. To maximize their effectiveness, a multi-stakeholder collaborative framework involving local governments, research institutions, and communities is crucial. Providing financial incentives for community-led conservation projects will further ensure that adaptive strategies are not only scientifically sound but also effectively implemented at the local level.

### 4.4. Limitations and Future Research Directions

This study offered a robust assessment of ecological vulnerability in the TENS. Our primary strength lies in applying the IPCC framework to long-term data. This approach successfully revealed critical spatiotemporal patterns. We identified distinct latitudinal and longitudinal vulnerability gradients. We also pinpointed nonlinear temporal dynamics and trend reversals linked to conservation efforts. These findings provide a solid foundation for adaptive management. However, several limitations should be acknowledged to guide future research.

First, the framework omits soil-related factors, such as moisture, organic matter, and erosion potential. These are vital in high-altitude areas with freeze–thaw cycles. This may underestimate vulnerability in wetlands and grasslands, where soil degradation amplifies sensitivity and reduces resilience. Second, the 1 km spatial resolution of datasets may miss fine-scale heterogeneity in complex topography with steep elevations (750–6500 m). This could overgeneralize patterns in transitional zones and hotspot migrations. Third, the focus on historical data ignores future climate projections, like intensified temperature and precipitation variability under IPCC scenarios. This limits proactive conservation planning amid accelerating changes.

Future research should address these gaps. Integrate soil metrics using hydrological models or remote sensing soil moisture indices for a holistic view. Use higher-resolution data, such as sub-kilometer NDVI from Sentinel-2 or downscaled climate models, to capture microscale dynamics. Incorporate CMIP 6 scenarios in dynamic models to forecast vulnerability trajectories. These steps will refine adaptive strategies and support IPCC-aligned science in climate-sensitive regions.

## 5. Conclusions

This study applied the IPCC exposure–sensitivity–resilience framework to NDVI, temperature, and precipitation data from 1983 to 2022 to assess the spatiotemporal dynamics of ecological vulnerability in the TENS. The results reveal pronounced spatial heterogeneity, with vulnerability increasing from south to north along a latitudinal gradient and forming a V-shaped pattern longitudinally due to differing monsoon influences. High-altitude areas show greater exposure to frost, freeze–thaw cycles, and shortened growing seasons. Ecosystem types moderate vulnerability in distinct ways. Wetlands remain the most fragile because their stability depends on hydrological balance, whereas forests exhibit the highest resilience through structural and biological diversity. Temporally, vulnerability displays nonlinear fluctuations with phases of significant decline, increase, decline and stabilization from 1985 to 2020. That was shaped jointly by climate variability and conservation actions, including ecological restoration and desertification control programs implemented in the late 2000s. The observed migration paths of vulnerability trends highlight the need for adaptive management and continuous monitoring. By integrating spatial and temporal patterns with ecosystem-specific vulnerability factors, this study provides a practical framework for conservation planning in TENS and similar ecologically sensitive regions. Future work should incorporate soil properties, finer-scale environmental data, and climate projections to improve model accuracy and support scenario-based adaptive strategies.

## Data Availability

The raw data supporting the conclusions of this article will be made available by the authors on request.

## Supplementary Materials

The following supporting information can be downloaded at: https://www.mdpi.com/article/10.3390/biology14111625/s1, Figure S1: Variations in VI and its components EI, SI, and RI with latitude, longitude, and altitude based on 2088 sampled point data. (a–c) EI; (d–f) SI; (g–i) RI; (j–l) VI. Left column: versus latitude; middle column: versus longitude; right column: versus altitude; Figure S2: Spatial patterns of VI with significant decreasing trends across four phases (a–d), with significant increasing trends across four phases (e,f); Figure S3: Relationship between annual average temperature (a), annual aridity index (AI) (b) with latitude, and the trend of vulnerability index (VI) with annual average temperature alongside latitude (c). The temperature, aridity index, and VI were calculated by averaging original sampling data at 1° latitude intervals. The annual aridity index (AI) data were from the National Earth System Science Data Center (https://www.geodata.cn/data/datadetails.html?dataguid=188606016270010&docid=126, accessed on 15 January 2025, as same in Figures S4 and S5); Figure S4: Relationship between annual average temperature (a), annual aridity index (AI) (b) with longitude, the trend of vulnerability index (VI) with annual average temperature alongside longitude (c), and the monsoon domain in the TENS region (d), as provided by the IPCC (https://ipcc-browser.ipcc-data.org); Figure S5: The variations in annual average temperature and annual aridity index from 1985 to 2020; Figure S6: The main geomorphic types within the TENS region. The spatial distribution data of geomorphic types were sourced from the “Geological Map of the People’s Republic of China (1:1,000,000)”; Table S1: Percentage of areas showing decreasing trends (DT), significant decreasing trends (SDT), increasing trends (IT) and significant increasing trends (SIT) in VI during four different phases; Table S2: Area proportions of different conversion types in VI trends; Table S3: Area proportions of geomorphic types among the range of the D-I-D-I pattern.

## Abbreviations

The following abbreviations are used in this manuscript:

TENS	the terrestrial ecosystems of Northwestern Sichuan
MAT	mean annual temperature
AP	annual precipitation
AI	aridity index
VI	vulnerability index
EI	exposure index
SI	sensitivity index
RI	resilience index
