# Supplementary material for: Spatiotemporal Dynamics of Ecological Vulnerability to Climate Change in Northwestern Sichuan’s Terrestrial Ecosystems of China: Conservation Implications
Source: Biology (Basel). 2025 Nov 19;14(11):1625. doi: 10.3390/biology14111625 (PMC12650117; doi:10.3390/biology14111625)
Supplement: Supplementary file 1 [file biology-14-01625-s001.zip › biology-3956519-supplementary.pdf]

Spatiotemporal Dynamics of Ecological Vulnerability to Climate Change in Northwestern Sichuan's Terrestrial Ecosystems of China: Conservation Implications

Cuicui Jiao *, Xiaobo Yi, Ji Luo, Ying Wang, Yuanjie Deng, Jiangtao Gou and Danting Luo

School of Economics, Sichuan University of Science & Engineering, Yibin 644000, China; abobobi@163.com (X.Y.); steelseek@suse.edu.cn (J.L.); hywangying@suse.edu.cn (Y.W.); ecodyj@suse.edu.cn (Y.D.); 18227618298@163.com (J.G.); 13683404454@163.com (D.L.)

* Correspondence: jiaocui_cui@163.com

Figure S1 Variations in VI and its components EI, SI, and RI with latitude, longitude, and altitude based on 2088 sampled point data. (a – c) EI; (d – f) SI; (g – i) RI; (j – l) VI. Left column: versus latitude; middle column: versus longitude; right column: versus altitude.

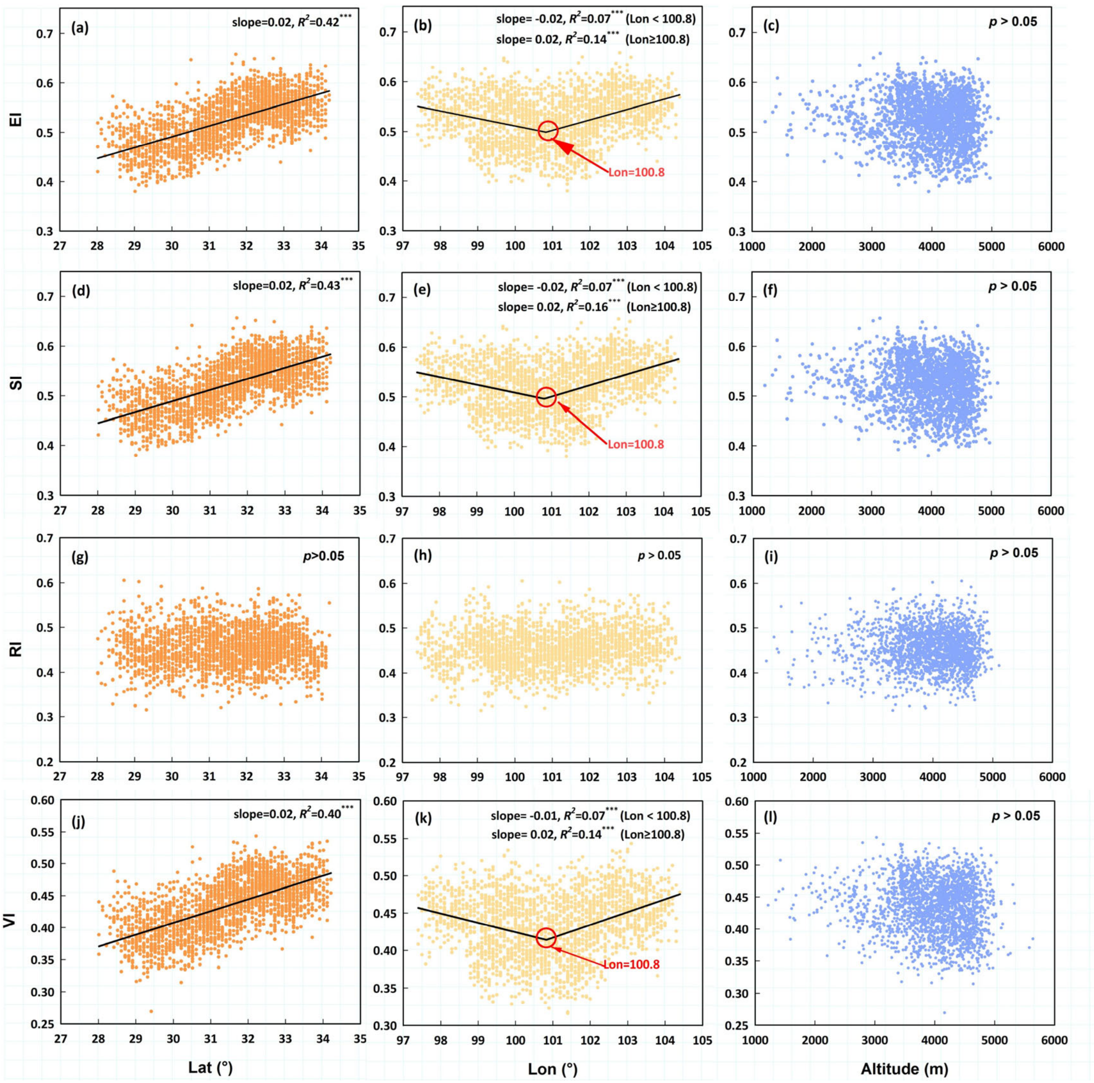

Figure S2 Spatial patterns of VI with significant decreasing trends across four phases (a-d), with significant increasing trends across four phases (e-f)

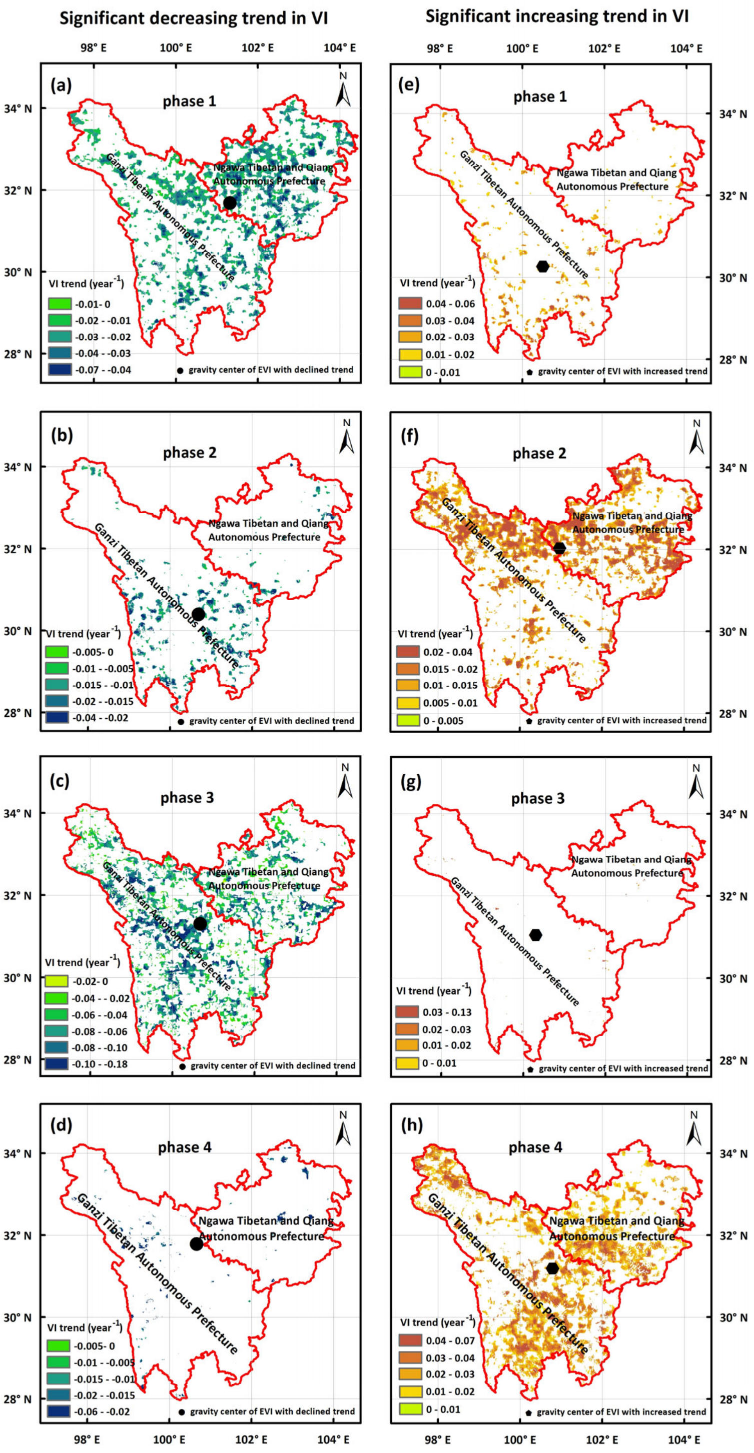

Figure S3 Relationship between annual average temperature (a), annual aridity index (AI) (b) with latitude, and the trend of vulnerability index (VI) with annual average temperature alongside latitude (c). The temperature, aridity index, and VI were calculated by averaging original sampling data at 1° latitude intervals. The annual aridity index (AI) data were from the National Earth System Science Data Center (https://www.geodata.cn/data/datadetails.html?dataguid=188606016270010&docid=126, as same in Figures S4 and S5)

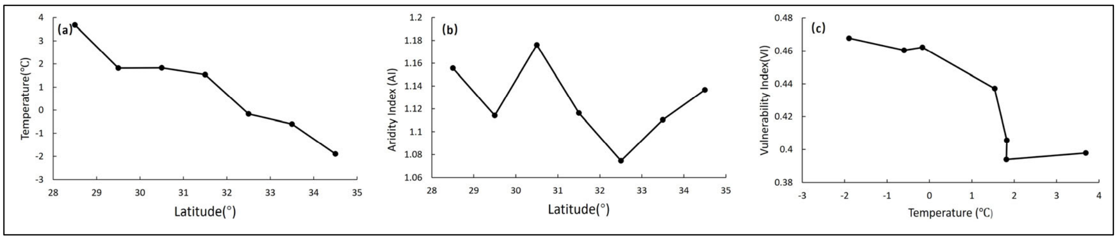

Figure S4 Relationship between annual average temperature (a), annual aridity index (AI) (b) with longitude, the trend of vulnerability index (VI) with annual average temperature alongside longitude (c), and the monsoon domain in the TENS region (d), as provided by the IPCC (https://ipcc-browser.ipcc-data.org)

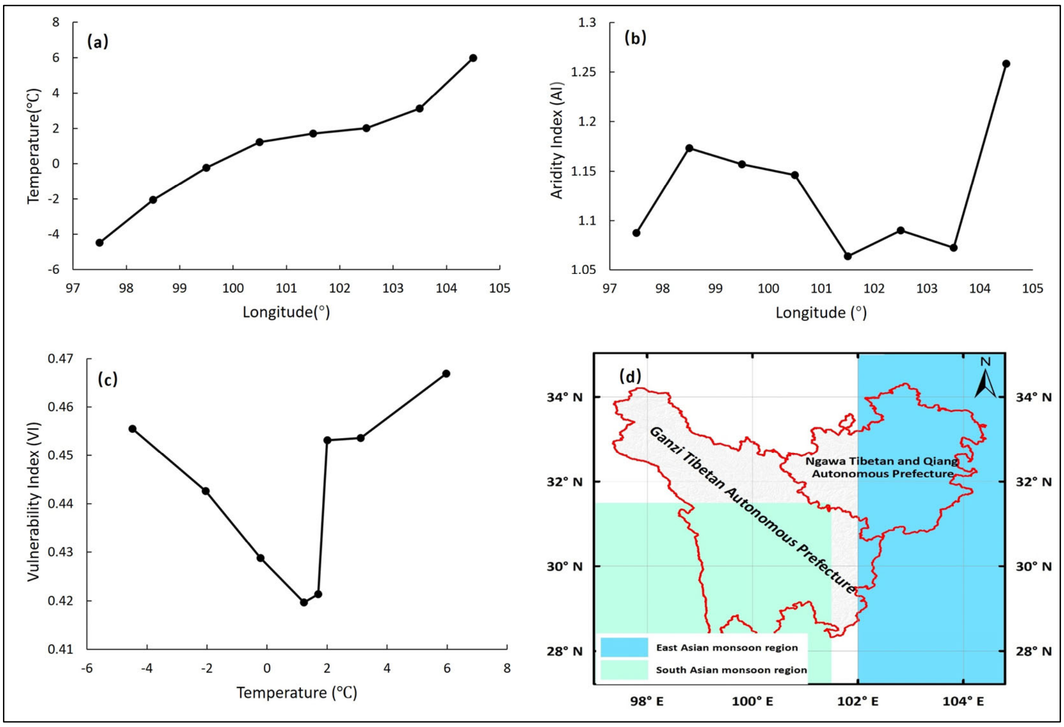

Figure S5 The variations of annual average temperature and annual aridity index from 1985 to 2020

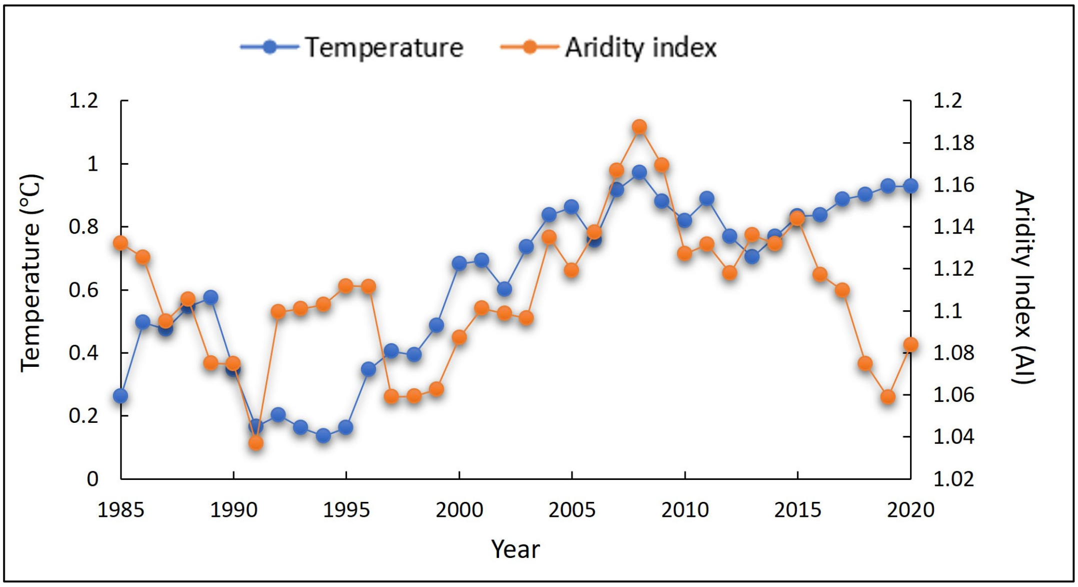

Figure S6 The main geomorphic types within the TENS region. The spatial distribution data of geomorphic types were sourced from the "Geological Map of the People's Republic of China (1:1,000,000)"

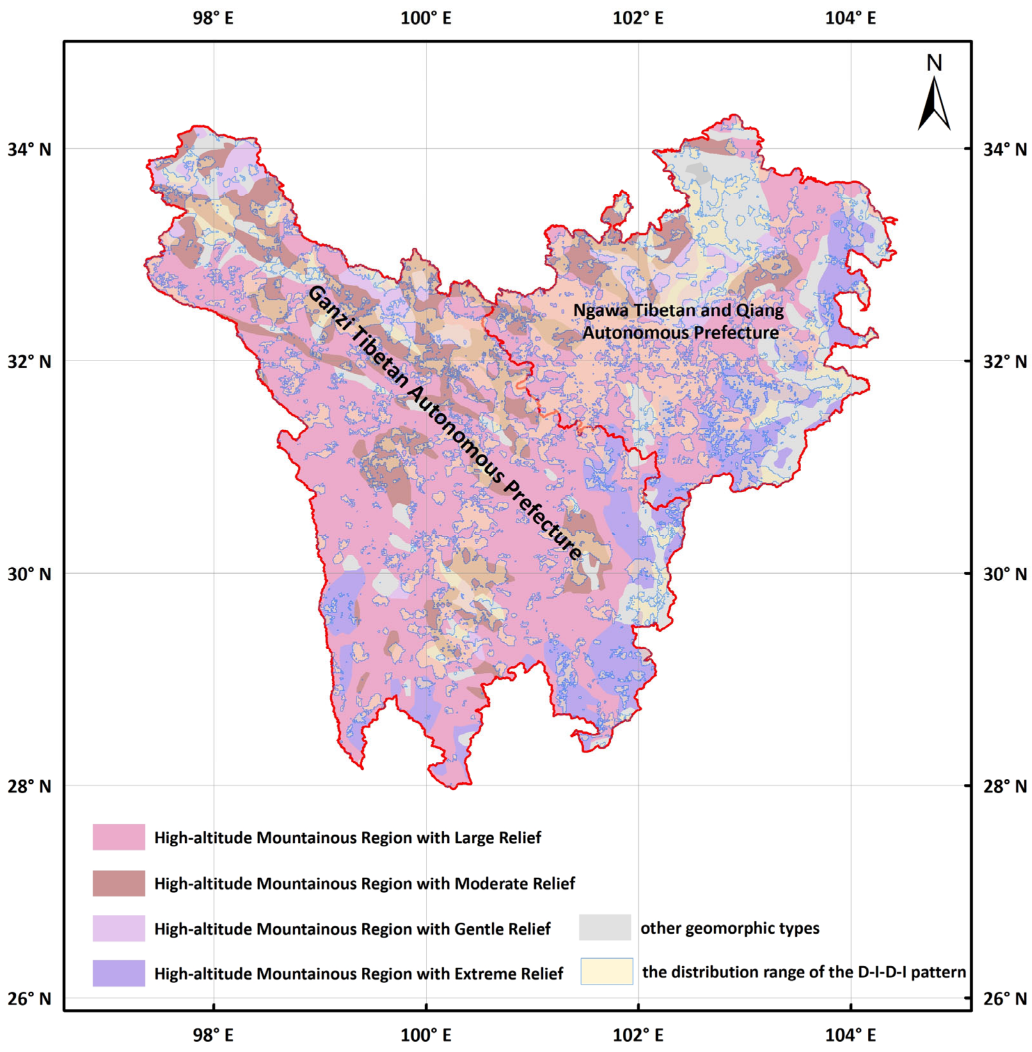

Table S1 Percentage of areas showing decreasing trends (DT), significant decreasing trends (SDT), increasing trends (IT) and significant increasing trends (SIT) in VI during four different phases

	DT (%)	SDT (%)	IT (%)	SIT (%)
1985-1994	69.06	27.76	30.94	4.76
1994-2008	31.17	7.23	68.83	31.43
2008-2011	93.96	24.89	6.04	0.15
2011-2020	19.67	1.38	80.33	33.31

Table S2 Area proportions of different conversion types in VI trends

	type	area proportion (%)
1	D-D-D-D	1.24
2	D-D-D-I	9.86
3	D-D-I-D	0.40
4	D-D-I-I	0.81

5	D-I-D-D	7.87
6	D-I-D-I	34.62
7	D-I-I-D	0.61
8	D-I-I-I	0.76
9	I-I-I-I	0.16
10	I-I-I-D	0.17
11	I-I-D-D	1.86
12	I-I-D-I	10.19
13	I-D-D-D	1.03
14	I-D-D-I	7.76
15	I-D-I-I	0.74
16	I-D-I-D	0.34
17	No trend	21.59

Table S3 Area proportions of geomorphic types among the range of the D-I-D-I pattern

	geomorphic type	area proportion (%)
1	High-altitude Mountainous Region with Large Relief	45.80
2	High-altitude Mountainous Region with Moderate Relief	21.99
3	High-altitude Mountainous Region with Extreme Relief	9.93
4	High-altitude Mountainous Region with Gentle Relief	7.30
